# Inconsistent Increase in Age at Respiratory Syncytial Virus Hospitalization of Children Aged <2 Years During the Severe Acute Respiratory Syndrome Coronavirus 2 Pandemic: A Retrospective Multicenter Study in 4 European Countries

**DOI:** 10.1093/infdis/jiae292

**Published:** 2024-06-24

**Authors:** Eline R Harding, Joanne G Wildenbeest, Terho Heikkinen, Ana Dacosta-Urbieta, Federico Martinón-Torres, Steve Cunningham, Kate Templeton, Louis J Bont, Marie-Noëlle Billard, PROMISE investigators, Eline Harding, Marie-Noëlle Billard, Joanne Wildenbeest, Louis Bont, Andrew Pollard, Ana Dacosta-Urbieta, Federico Martinón-Torres, Terho Heikkinen, Steve Cunningham, Kate Templeton, Harish Nair, Peter Openshaw, Philippe Beutels, Hannah Nohynek, Anne Teirlinck, John Paget, Leyla Kragten, Carlo Giaquinto, Javier Diez-Domingo, Rafael Mikolajczyk, Gael Dos Santos, Tin Tin Htar, Jeroen Aerssens, Charlotte Vernhes, Rolf Kramer, Veena Kumar, Bahar Ahani, Eva Molero

**Affiliations:** Department of Pediatric Infectious Diseases and Immunology, Wilhelmina Children's Hospital, University Medical Center Utrecht, Netherlands; Department of Pediatric Infectious Diseases and Immunology, Wilhelmina Children's Hospital, University Medical Center Utrecht, Netherlands; Department of Pediatrics, University of Turku and Turku University Hospital, Finland; Translational Paediatrics and Infectious Diseases, Paediatrics Department, Hospital Clínico Universitario de Santiago de Compostela, Santiago de Compostela, Spain; Genetics, Vaccines and Infections Research Group, Instituto de Investigación Sanitaria de Santiago, University of Santiago, Santiago de Compostela, Spain; Centro de Investigación Biomédica en Red de Enfermedades Respiratorias, Instituto de Salud Carlos III, Madrid, Spain; Translational Paediatrics and Infectious Diseases, Paediatrics Department, Hospital Clínico Universitario de Santiago de Compostela, Santiago de Compostela, Spain; Genetics, Vaccines and Infections Research Group, Instituto de Investigación Sanitaria de Santiago, University of Santiago, Santiago de Compostela, Spain; Centro de Investigación Biomédica en Red de Enfermedades Respiratorias, Instituto de Salud Carlos III, Madrid, Spain; Department of Child Life and Health, Centre for Inflammation Research, University of Edinburgh, Edinburgh, United Kingdom; Department of Medical Microbiology, Royal Infirmary, NHS Lothian, Edinburgh, United Kingdom; Department of Pediatric Infectious Diseases and Immunology, Wilhelmina Children's Hospital, University Medical Center Utrecht, Netherlands; ReSViNET Foundation, Julius Clinical, Zeist, Netherlands; Department of Pediatric Infectious Diseases and Immunology, Wilhelmina Children's Hospital, University Medical Center Utrecht, Netherlands

**Keywords:** RSV, COVID-19 pandemic, children, epidemiology, hospitalization

## Abstract

**Background:**

The severe acute respiratory syndrome coronavirus 2 (SARS-CoV-2) pandemic disrupted respiratory syncytial virus (RSV) seasonality. To optimize the use and evaluation of RSV infant immunization strategies, monitoring changes in RSV epidemiology is essential.

**Methods:**

Hospitalizations for acute respiratory infections (ARIs) and RSV-coded ARI in children <2 years were extracted in 4 European hospitals, according to predefined case definitions (*International Classification of Diseases, Tenth Revision* codes). Prepandemic RSV seasons (2017–2018 to 2019–2020) were compared to 2021–2022 and 2022–2023.

**Results:**

In 2021–2022 and 2022–2023, the peak number of RSV hospitalizations was higher than prepandemic peaks after short periods of RSV circulation, and lower than prepandemic peaks after long periods of RSV circulation. A greater proportion of RSV hospitalizations occurred in children 1 to <2 years in 2021–2022 in the Netherlands (18% vs 9%, *P* = .04). No increase in age was observed elsewhere. High-risk children represented a greater proportion of RSV hospitalizations during the pandemic. The proportion of pediatric intensive care unit admissions did not increase.

**Conclusions:**

A decrease in population immunity has been linked to older age at RSV hospitalization. We did not observe an increase in age in 3 of the 4 participating countries. Broad age categories may have prevented detecting an age shift. Monitoring RSV epidemiology is essential as Europe implements RSV immunization.

## Teaser

During the SARS-CoV-2 pandemic, respiratory syncytial virus hospitalization patterns in children aged <2 years changed. Although a shift to older age was seen in the Netherlands, we did not observe an increase in age in the other 3 participating countries in Europe.

Acute respiratory infections (ARIs) cause high morbidity and mortality in young children worldwide [1]. Respiratory syncytial virus (RSV) is the most common virus in children <5 years of age with acute lower respiratory tract infection, with the highest burden found in children aged <1 year [2, 3]. In 2019, RSV contributed to 3.6 million hospital admissions and 26 000 in-hospital deaths in children aged <5 years worldwide [3]. In a European prospective birth cohort study, 1.8% of healthy term-born infants were hospitalized for RSV, of whom 5.5% were admitted to the pediatric intensive care unit (PICU) [4]. Although deaths due to RSV are rare in high-income countries, RSV causes important annual surges of hospitalizations [5].

Before the severe acute respiratory syndrome coronavirus 2 (SARS-CoV-2) pandemic, RSV was seasonal in most geographic regions, causing winter epidemics in temperate countries. In tropical countries, RSV seasonality showed more variations [6]. In Europe, differences in the timing of the RSV season were observed between countries with a West to East gradient [7]. In the United Kingdom (UK), Spain, and the Netherlands, the RSV season started in November–December and peaked in December–January [7, 8]. In Finland, RSV seasonality showed a biennial pattern, characterized by a mild and short season, alternating with an extended season during the following winter [8].

When SARS-CoV-2 was declared a pandemic in March 2020 and nonpharmaceutical interventions (NPIs) were implemented to slow down its spread, RSV circulation in Europe dropped [9]. Subsequently, RSV seasonality was severely disrupted. During the winter of 2020–2021, unusually low RSV activity was detected [10]. RSV reemerged in 2021 causing out-of-season epidemics during the summer in various countries, including the UK, Spain, and the Netherlands [10–13]. In contrast, RSV was not observed in Finland until the end of summer 2021 [14].

In addition to the time shifts of RSV epidemics, changes in patient characteristics have been reported during the SARS-CoV-2 pandemic. Multiple countries reported an older median age at RSV hospitalization [15–21] or a larger increase in the incidence of RSV hospitalizations in children aged 1–5 years compared with infants [11, 22]. Accordingly, a recent systematic analysis found an increase in the proportion of RSV hospitalizations in children aged 1 to <2 years during the SARS-CoV-2 pandemic (until March 2022) -compared with prepandemic seasons, after adjusting for testing practices [23]. There were also conflicting reports regarding a possible increase in disease severity causing more PICU admissions during the first reemergence of RSV [19, 24, 25].

Monitoring RSV circulation patterns in Europe is critically important to inform hospital bed capacity planning during RSV peaks and to optimize RSV immunization programs. As new infant immunization strategies are not expected to provide long-term immunity, timing of immunization is important [26, 27]. Understanding changes in RSV circulation patterns will also facilitate evaluation of RSV immunization programs. Although multiple studies have described changes in RSV epidemiology during the SARS-CoV-2 pandemic, most were conducted in a single country, did not include prepandemic seasons, or compared results obtained with different methods or surveillance systems [28, 29].

This study aimed to describe the differences in the number and the characteristics of ARI and RSV hospitalizations in children aged <2 years in 4 European hospitals during 3 prepandemic seasons (2017–2020) and 2 pandemic seasons (2021–2023). To our knowledge, this is the first study to document changes in RSV seasonality in 4 European countries using the same case definitions and inclusion criteria.

## METHODS

This retrospective observational multicenter study was conducted by the PROMISE (Preparing for RSV Immunization and Surveillance in Europe) Consortium. The aim was to describe changes in RSV epidemiology during the SARS-CoV-2 pandemic in 4 hospitals from different European countries that previously participated in the RESCEU (REspiratory Syncytial virus Consortium in EUrope) birth cohort study [4]: Hospital Clínico Universitario de Santiago de Compostela, Servicio Galego de Saúde (Spain), Royal Hospital for Children and Young People, Edinburgh (Scotland), Wilhelmina Children's Hospital, Utrecht (the Netherlands), and Turku University Hospital (Finland). Although these hospitals were tertiary care centers with a PICU and likely not representative of the entire country, country names are used hereafter for simplicity.

The number and characteristics of monthly ARI and RSV hospitalizations between July 2017 and May 2023 in children aged <2 years were collected retrospectively. The 2 seasons during the SARS-CoV-2 pandemic (2021–2022 and 2022–2023) were compared to prepandemic seasons (2017–2018 to 2019–2020). The sites tested all children aged <2 years admitted with ARI for RSV before and during the pandemic. During the RESCEU birth cohort study (2017–2020), RSV test results were found for >90% of respiratory hospitalizations at these sites [4]. Testing policies per site and RSV season (Supplementary Table 1) were used to support interpretation. We did not correct for testing policies as no substantial change was reported during the study period.

ARI hospitalizations were defined as admissions to the ward or PICU, with at least 1 respiratory *International Classification of Diseases, Tenth Revision* (*ICD-10*) code as the primary or secondary code (Supplementary Table 2). RSV hospitalizations were defined as ARI hospitalizations with an RSV-specific *ICD-10* code and/or a positive RSV test result. The hospitalizations were stratified by age group (0 to <1 year and 1 to <2 years at admission) and according to PICU admission. In Spain and the Netherlands, additional data about sex, preterm birth (gestational age <37 weeks), and the presence of at least 1 other comorbidity (ie, congenital heart disease, bronchopulmonary disease, chronic or congenital lung disease, cystic fibrosis, immunodeficiency, genetic or chromosomal disease, and neuromuscular disorder) were available for analysis. We considered children born preterm or with at least 1 comorbidity to be at high risk for RSV hospitalization.

The 3 prepandemic seasons (2017–2018, 2018–2019, and 2019–2020) were defined as 12-month periods centered around the peak from July to June in Scotland, Spain, and the Netherlands and from August to July in Finland to accommodate Finland's longer seasons. Little to no RSV activity was observed during the 2020–2021 RSV season in most European countries. Subsequently, out-of-season epidemics were reported [10]. Thus, we defined two 12-month periods of RSV during the pandemic (2021–2022 and 2022–2023) that were tailored to the timing of the RSV peaks observed in each country (Table 1). The pandemic RSV seasons were defined to be centered around the peaks and have the cut-off between the 2021–2022 and the 2022–2023 season in the months with the smallest number of RSV hospitalizations. The peak of each RSV season was defined as the month with the highest number of RSV hospitalizations.

**Table 1.** Start and End Month of the Respiratory Syncytial Virus Season, 2017–2018 to 2022–2023

Site	2017–2018	2018–2019	2019–2020	2021–2022	2022–2023
Scotland	July 2017–June 2018	July 2018–June 2019	July 2019–June 2020	May 2021–Apr 2022	May 2022–Apr 2023
Finland	Aug 2017–July 2018	Aug 2018–July 2019	Aug 2019–July 2020	June 2021–May 2022	June 2022–May 2023
Spain	July 2017–June 2018	July 2018–June 2019	July 2019–June 2020	Apr 2021–Mar 2022	Apr 2022–Mar 2023
The Netherlands	July 2017–June 2018	July 2018–June 2019	July 2019–June 2020	Apr 2021–Mar 2022	Apr 2022–Mar 2023

Descriptive statistics were used to summarize clinical data. Differences between pre- and postpandemic proportions were calculated by χ^2^ or Fisher test.

### Ethics

An institutional review board waiver was obtained at each site as this study exclusively reused aggregated data routinely collected in hospitals.

## RESULTS

**Figure 1.**
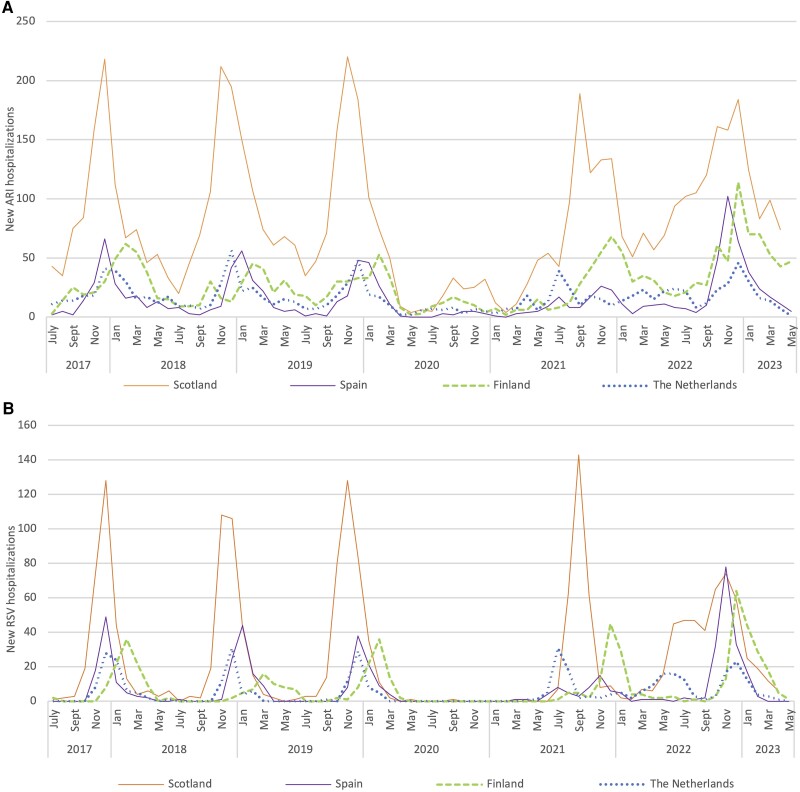
Number of new acute respiratory infections (ARI; *A*) and respiratory syncytial virus (RSV; *B*) hospitalizations per calendar month in children <2 years of age in Scotland, Spain, Finland, and the Netherlands. Spain collected data up to and including March 2023. Scotland collected data up to and including April 2023.

Annual peaks of RSV hospitalizations corresponded to peaks of ARI hospitalizations (Figure 1). During prepandemic seasons (2017–2020), the number of ARI and RSV hospitalizations fluctuated slightly between seasons in each country but varied between countries (Table 2). The average number of ARI hospitalizations per season varied between 191 in Spain (range, 168–208) and 1042 in Scotland (range, 959–1168). During the same period, the average number of RSV hospitalizations varied between 60 in the Netherlands (range, 52–74) and 322 in Scotland (range, 303–361). On average, the proportion of ARI hospitalizations that tested RSV positive or had a RSV-specific diagnosis was between 27% (range, 20%–32%) in Finland and 46% (range, 42%–49%) in Spain.

**Table 2.** Number of Acute Respiratory Infections and Respiratory Syncytial Virus Hospitalizations in Children <2 Years of Age per Season—Scotland, Spain, Finland, and the Netherlands, 2017–2018 to 2022–2023

Country	2017–2018	2018–2019	2019–2020	Average Pre-pandemic	2021–2022		2022–2023	
	No. (% RSV/ARI)	No. (% RSV/ARI)	No. (% RSV/ARI)	No. (% RSV/ARI)	No. (% RSV/ARI)	Relative Change	No. (% RSV/ARI)	Relative Change
Scotland								
ARI hospitalizations	999	1168	959	1042	1067	1.02	1373	1.32
RSV hospitalizations	303 (30.3%)	303 (25.9%)	361 (37.6%)	322 (30.9%)	306 (28.7%)	0.95	455 (33.1%)	1.41
Spain								
ARI hospitalizations	208	198	168	191	140	0.73	343	1.80
RSV hospitalizations	88 (42.3%)	96 (48.5%)	80 (47.6%)	88 (46.1%)	56 (40%)	0.64	170 (49.6%)	1.93
Finland								
ARI hospitalizations	353	282	262	299	390	1.30	600	2.01
RSV hospitalizations	99 (28.0%)	55 (19.5%)	85 (32.4%)	80 (26.8%)	111 (28.5%)	1.39	181 (30.2%)	2.26
The Netherlands								
ARI hospitalizations	246	222	175	214	210	0.98	260	1.21
RSV hospitalizations	74 (30.1%)	52 (23.4%)	55 (31.4%)	60 (28.0%)	80 (38.1%)	1.33	121 (46.5%)	2.02

Abbreviations: ARI, acute respiratory infection; RSV, respiratory syncytial virus.

During the pandemic, there were 2 peaks of ARI hospitalizations at all sites that matched the timing of RSV peaks (Figure 1). Compared to prepandemic seasons, the number of ARI hospitalizations increased at all sites in 2022–2023, ranging from 1.2 times higher in the Netherlands to 2.0 times higher in Finland (Table 2).

Compared to prepandemic seasons, the number of RSV hospitalizations in 2021–2022 was 1.3 times higher in the Netherlands (80 vs 60) and 1.4 times higher in Finland (111 vs 80). In 2022–2023, the number of RSV hospitalizations increased compared to prepandemic seasons at all sites, ranging from 1.4 times higher in Scotland (455 vs 322) to 2.3 times higher in Finland (181 vs 80) (Table 2).

During the 3 prepandemic seasons, RSV showed clear seasonality at all sites with an annual peak during the fall or winter (Figure 1). The number of RSV hospitalizations peaked first in Scotland (November–December), approximately 1 month earlier than in the Netherlands (December), 2 months earlier than in Spain (December–January), and 3 months earlier than in Finland (February–March). In Finland, RSV peaked in February (2017–2018 and 2019–2020) or in March (2018–2019) with the 2018–2019 season lasting longer, with hospitalizations until June.

**Figure 2.**
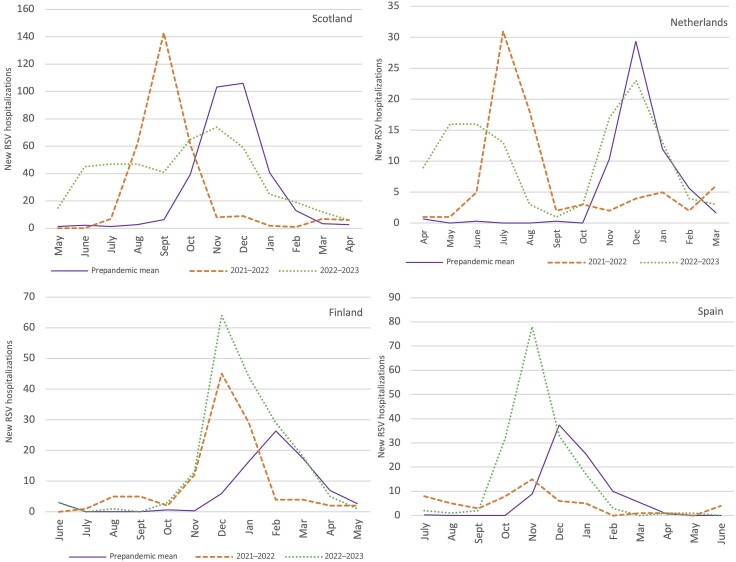
Number of new respiratory syncytial virus (RSV) hospitalizations per calendar month in Scotland, the Netherlands, Finland, and Spain in 2020–2021, 2021–2022, 2023–2023, and prepandemic seasons (2017–2018 to 2019–2020). The prepandemic mean is the monthly average number of RSV hospitalizations from July 2017 to April 2020 in Scotland, from July 2017 to March 2020 in the Netherlands, from July 2017 to May 2020 in Finland, and from July 2020 to June 2020 in Spain.

After SARS-CoV-2 was declared a pandemic in March 2020 [9], the number of RSV hospitalizations dropped rapidly (Figure 1). No RSV hospitalizations in children <2 years were observed before spring 2021, which included the expected period for the 2020–2021 RSV season. The RSV peak at reemergence (referred to as the 2021–2022 season in this article) was shifted at all sites (Figure 2). Two different circulation patterns of RSV were observed. In Spain and the Netherlands, where the first RSV hospitalizations were observed in March or April 2021, the 2021–2022 RSV season was characterized by a summer peak followed by a prolonged period of RSV circulation. In these countries, the peak at reemergence was approximately 5 months early compared with prepandemic peaks (July 2021). This initial peak was followed by a second peak in November 2021 in Spain and by a prolonged period of circulation through winter 2021–2022 in the Netherlands. In contrast, in Finland and Scotland where the first RSV hospitalizations were observed in July 2021, the 2021–2022 RSV season was characterized by a single peak, earlier than prepandemic peaks. The peak was 2–3 months early in Finland (December 2021) and 1–2 months early in Scotland (September 2021) compared to prepandemic peaks. The number of RSV hospitalizations during the peak month was lower than prepandemic peaks in Spain (15 vs 44) and of similar amplitude in the Netherlands (31 vs 29), whereas the peak was higher than prepandemic peaks in Scotland (143 vs 121) and Finland (45 vs 29).

In 2022–2023, the shift in timing of peak number of RSV hospitalizations was less than in 2021–2022 (Figure 2). Although the number of RSV hospitalizations started to increase several months early in Scotland and the Netherlands (March 2022), the timing of the peak was comparable to prepandemic seasons (November 2022 in Scotland and December 2022 in the Netherlands). In contrast, in Finland and Spain, the number of RSV hospitalizations started to increase in October 2022, as in prepandemic seasons. However, the peak of the 2022–2023 season was 1–2 months early in Spain (November) and 2–3 months early in Finland (December). The number of RSV hospitalizations during the peak month in 2022–2023 was lower than prepandemic peaks in Scotland (74 vs 121) and the Netherlands (23 vs 29) and higher in Spain (78 vs 44) and Finland (64 vs 29).

Most RSV hospitalizations occurred in children <1 year of age in all countries and seasons (Table 3). In 2021–2022, a higher proportion of RSV hospitalizations occurred in children aged 1 to <2 years in the Netherlands compared to prepandemic seasons (18% vs 9%, *P* = .04). No change in the distribution of RSV hospitalizations between age groups (<1 and 1 to <2 years) was observed at other sites, or during the 2022–2023 season.

**Table 3.** Characteristics of Respiratory Syncytial Virus–Coded Hospitalizations During Prepandemic Seasons (2017–2018 to 2019–2020) and in 2021–2022 and 2022–2023 in Finland, Scotland, Spain, and the Netherlands

Characteristic	Scotland					The Netherlands					Spain					Finland				
	Pre-pandemic	2021–2022		2022–2023		Pre-pandemic	2021–2022		2022–2023		Pre-pandemic	2021–2022		2022–2023		Pre-pandemic	2021–2022		2022–2023	
	No. (%)	No. (%)	*P* Value^a^	No. (%)	*P* Value^a^	No. (%)	No. (%)	*P* Value^a^	No. (%)	*P* Value^a^	No. (%)	No. (%)	*P* Value^a^	No. (%)	*P* Value^a^	No. (%)	No. (%)	*P* Value^a^	No. (%)	*P* Value^a^
Total	967	306		455		181	80		121		264	56		170		239	111		181	
Age at hospitalization																				
<1 y	810 (83.8%)	249 (81.4%)	.33	366 (80.4%)	.12	165 (91.2%)	66 (82.5%)	.**04**	108 (89.3%)	.58	233 (88.3%)	50 (89.3%)	.83	150 (88.2%)	.99	189 (79.1%)	85 (76.6%)	.60	134 (74.0%)	.22
1 to <2 y	157 (16.2%)	57 (18.6%)		89 (19.6%)		16 (8.8%)	14 (17.5%)		13 (10.7%)		31 (11.7%)	6 (10.7%)		20 (11.8%)		50 (20.9%)	26 (23.4%)		47 (26.0%)	
PICU admission																				
All	172 (17.8%)	37 (12.1%)	**.02**	55 (11.0%)	**.006**	104 (57.5%)	44 (55.0%)	.71	79 (65.3%)	.17	60 (22.7%)	12 (21.4%)	.83	22 (12.9%)	.**01**	27 (11.3%)	9 (8.1%)	.36	16 (8.8%)	.41
<1 y	139 (80.8%)	29 (78.4%)		43 (78.2%)		98 (94.2%)	38 (86.4%)		77 (97.5%)		50 (83.3%)	8 (66.7%)		16 (72.7%)		22 (81.5%)	7 (77.8%)		12 (75.0%)	
1 to <2 y	33 (19.2%)	8 21.6%)		12 (21.8%)		6 (5.8%)	6 (13.6%)		2 (2.5%)		10 (16.7%)	4 (33.3%)		6 (27.3%)		5 (18.5%)	2 (22.2%)		4 (25.0%)	
High-risk groups																				
At least 1	…	…		…		61 (33.7%)	40 (50.0%)	.**01**	71 (58.7%)	**<**.**001**	7 (2.7%)	10 (17.9%)	**<**.**001**	17 (10.0%)	.**001**	…	…		…	
Preterm birth	…	…		…		27 (44.3%)	15 (37.5%)		30 (42.3%)		6 (85.7%)	4 (40.0%)		11 (64.7%)		…	…		…	
Comorbidity	…	…		…		30 (49.2%)	21 (52.5%)		32 (45.1%)		1 (14.3%)	2 (20.0%)		3 (17.6%)		…	…		…	
Both	…	…		…		4 (6.6%)	4 (10.0%)		9 (12.7%)		0 (0%)	4 (40.0%)		3 (17.6%)		…	…		…	

Abbreviation: PICU, pediatric intensive care unit.

^a^ *P* value for comparison between the 2021–2022 or the 2022–2023 respiratory syncytial virus season with the prepandemic average. A *P* value <0.05 was considered statistically significant.

The overall proportion of RSV hospitalizations that required PICU admissions was higher in the Netherlands (59%) than in Finland (9%), Scotland (14%), and Spain (19%) (Table 3). Compared to prepandemic seasons, the proportion of PICU admissions among RSV hospitalizations was significantly lower in Scotland during the 2021–2022 (12% vs 18%, *P* = .02) and the 2022–2023 (11% vs 18%, *P* = .006) seasons, and in Spain during the 2022–2023 (13% vs 23%, *P* = .01) season. In Finland and the Netherlands, the proportion of PICU admissions was comparable to prepandemic seasons.

Information on preterm birth and comorbidity was available in Spain and the Netherlands (Table 3). Overall, high-risk children represented a larger proportion of RSV hospitalizations in the Netherlands (47%) than in Spain (10%). Compared to prepandemic seasons, the proportion of high-risk children among RSV hospitalizations increased in the Netherlands in 2021–2022 (50% vs 34%, *P* = .01) and 2022–2023 (59% vs 34%, *P* < .001). Despite small numbers of high-risk children reported in Spain (≤10 per season), the proportion of high-risk children was higher in 2021–2022 (18% vs 3%, *P* < .001) and 2022–2023 (10% vs 3%, *P* = .001).

## DISCUSSION

In this retrospective multicenter study, we described changes in the number and characteristics of monthly RSV hospitalizations during the SARS-CoV-2 pandemic in 4 European hospitals. During the pandemic, the peak number of RSV hospitalizations was higher than prepandemic peaks after short periods of RSV circulation and lower than prepandemic peaks after long periods of RSV circulation. An increase in the proportion of older children (1 to <2 years) was only observed in the Netherlands in 2021–2022 (18% vs 9%). The proportion of PICU admissions did not increase. High-risk children represented a larger proportion of RSV hospitalizations in 2021–2022 and 2022–2023 than in prepandemic seasons in Spain and the Netherlands.

We identified 2 distinct patterns of circulation during the 2021–2022 and 2022–2023 RSV seasons. In the Netherlands and Scotland in 2021–2022 and in Spain and Finland in 2022–2023, the peak number of RSV hospitalizations was higher than during prepandemic seasons and it followed a short period of RSV circulation before the peak. In contrast, in Spain in 2021–2022 and in Scotland and the Netherlands in 2022–2023, the peak in the number of RSV hospitalizations was lower than during prepandemic seasons and followed a longer period of RSV circulation before the peak. Although changes in testing practice could have explained these results, no major policy change was reported. Also, similar RSV pandemic circulation patterns have been previously reported in these countries [11–14, 17, 30, 31]. European borders were not completely closed during the SARS-CoV-2 pandemic. Thus, these 2 RSV circulation patterns likely resulted from differences in local NPI policies and population behaviors that resulted in more or less favorable circumstances for RSV spread [32].

While we observed a larger proportion of children 1 to <2 years among RSV hospitalizations in the Netherlands in 2021–2022, this was not observed at other sites or in 2022–2023. An increase in age at RSV hospitalization in children <2 years has been previously reported in a nationwide study from the Netherlands (132 days in 2021–2022; 69 days during prepandemic seasons) and in a single-center study from Spain (4.9 months in spring and summer 2021; 3 months during prepandemic seasons) [13, 18]. In the UK, while no increase in age at RSV hospitalization was found in children aged <1 year, the median age of RSV-related emergency department visits was higher during summer 2021 than during prepandemic seasons (1.8 years and 0.3 year, respectively) [17]. In Finland, the peak in the number of RSV-positive cases in children 0–4 years increased in surveillance data, but age was not further stratified [33]. The age categories in our study (<1 year or 1 to <2 years) may have been too broad and statistical power too limited to allow detection of an age shift. While intensified testing in older children could result in an apparent older age at admission, a recent systematic analysis corrected for testing practices reported an increased proportion of children aged 1 to <2 years among RSV hospitalizations during the pandemic [23]. The main hypothesis explaining changes in RSV epidemiology during the SARS-CoV-2 pandemic is a decrease in population immunity due to a prolonged absence of exposure to RSV, referred to as immunity debt [10]. Susceptible individuals, including older RSV-naive children, accumulated in 2020 and 2021 through new births and waning immunity [34, 35]. Accordingly, an increase in age at RSV hospitalization would be expected, despite a smaller risk of RSV severe disease with older age [4]. Waning immunity has been identified as an important parameter to replicate pandemic RSV circulation patterns [13, 36]. Yet, other factors may have played a role. The duration and stringency of NPIs and the frequency of viral importation may have contributed to the shape and timing of RSV epidemics since the pandemic [13, 36]. Low levels of RSV circulation in 2021 before the peak could have contributed to filling the immunity gap [37]. The proportion of RSV-naive infants may have returned to prepandemic levels before the 2022–2023 RSV season, explaining why we observed no shift in age in 2022–2023 [34]. Overall, this study was not designed to confirm the existence of an immunity gap.

Compared to prepandemic seasons, we found a larger proportion of high-risk children among RSV hospitalizations in 2021–2022 and 2022–2023 in Spain and the Netherlands. Although in Spain ≤10 high-risk children were hospitalized annually, the increase was consistent in both season and both sites. As the increased risk of severe RSV disease in high-risk children persists at older age [38], high-risk children may have been more affected by waning immunity and the immunity gap [39]. Overall, a larger proportion of hospitalizations was in high-risk children in the Netherlands (34%–59%) than in Spain (3%–18%). In previous studies, 20%–30% of RSV hospitalizations <12 months occurred in high-risk children [38]. Using *ICD-10* diagnostic codes of ARI hospitalizations to identify high-risk groups might have resulted in missed cases in Spain while in the Netherlands, all *ICD-10* codes for comorbidities in any hospitalization since birth were available. Differences or changes in palivizumab indication could lead to differences in the proportion of high-risk children. In both countries, palivizumab was recommended mainly for children born <32 weeks of gestational age or those with comorbidities and no change was made during the pandemic [40, 41].

Our results did not indicate an increase in the severity of RSV hospitalizations in children <2 years as we observed no increase in the proportion of RSV hospitalizations that required PICU admissions in 2021–2022 and 2022–2023. This observation was consistent with previous reports [22, 42]. A recent global systematic analysis that compared the occurrence of severe outcomes from RSV-associated hospitalizations and the in-hospital case-fatality ratio also reported no consistent changes in disease severity [23]. Other factors than RSV disease severity could have influenced the proportion of PICU admissions, including changes in bed capacity, PICU admission policies, or palivizumab eligibility criteria during the pandemic. Notably, the decrease in the proportion of PICU admissions among RSV hospitalizations in Scotland in 2021–2022 and 2022–2023 may be linked to the extension of eligibility criteria for palivizumab administration in high-risk infants from July 2021 [43]. In addition, a PICU outreach service was gradually expanded to support the management of borderline cases in ward areas or in high-dependency care settings in 2022. We cannot exclude that an undetected older age at admission could have resulted in less severe cases.

The main strength of this study is the predefined inclusion criteria and case definitions for ARI and RSV hospitalizations. We accounted for the disruption of RSV seasonality during the pandemic, by defining the 2021–2022 and 2022–2023 RSV season according to the timing of the RSV peaks. To ensure that the observed time trends reflected epidemiological changes, we collected information on RSV testing policies at all sites, which did not reveal significant changes. This study also has limitations. First, the use of *ICD-10* codes to identify RSV hospitalizations tends to underestimate the true burden of RSV disease [44]. Underestimating the number of RSV hospitalizations would not have impacted the time trends at each site unless coding practices changed. However, other respiratory viruses can cause similar clinical presentation to RSV and had different circulation patterns. While it was not possible to ascertain RSV test results in this study, children aged <2 years admitted with respiratory symptoms were systematically tested for RSV in all 4 hospitals included. Thus, coding practices were assumed to reflect RSV test results. This assumption is supported by the absence of RSV hospitalizations between May 2020 and April 2021 while rhinovirus—a known cause of bronchiolitis—was circulating and the timing of the peak of RSV hospitalizations in 2021–2022 and 2022–2023 that matched previous reports and surveillance data [11, 13, 14, 30, 31]. Second, the data were aggregated by calendar year and month, which limited the detection of the peak and the duration of the RSV season. However, our results are consistent with previously reported circulation patterns during the pandemic [11, 13, 14, 30, 31]. Third, we collected aggregated data for this study, including age in broad categories (<1 year and 1 to <2 years), which could have prevented detection of an age shift. Fourth, participating sites were not directly comparable, and countries have different healthcare systems. Although these differences limited comparisons between sites, they would not have impacted the time trends at each study site.

In conclusion, RSV circulation was largely disrupted at the 4 sites from April 2020 onward. While a decrease in population immunity has been suggested to result in older age at RSV hospitalization, we did not observe an older age at RSV hospitalization in 3 of the 4 participating sites. This study relied on the assumption that coding practice reflected RSV test results as respiratory hospitalizations in children <2 years were systematically tested in the 4 participating hospitals. To maximize the potential of electronic hospital records as real-world data in the context of evolving testing practices for respiratory pathogens, electronic systems should make positive and negative test results easy to extract. As European countries introduce the new RSV immunization strategies, it is essential to continue monitoring RSV epidemiology. Accounting for changes in seasonality will allow to maximize the benefits of RSV immunization programs.

## Supplementary Data

Supplementary materials are available at *The Journal of Infectious Diseases* online (http://jid.oxfordjournals.org/). Supplementary materials consist of data provided by the author that are published to benefit the reader. The posted materials are not copyedited. The contents of all supplementary data are the sole responsibility of the authors. Questions or messages regarding errors should be addressed to the author.

## Supplementary Material

jiae292_Supplementary_Data

## Notes

***Acknowledgments.*** We thank the local study teams for proving the site-specific data.

***Author contributions*.** M.-N. B., J. G. W., and L. J. B. designed the study. T. H., A. D., F. M.-T., S. C., K. T., E. R. H., and M.-N. B. collected data. E. R. H. and M.-N. B. analyzed and interpreted data. E. H. wrote the first draft. M.-N. B., L. J. B., J. W., T. H., A. D.-U., F. M.-T., and S. C. reviewed and commented on the manuscript. E. R. H., M. B., and J. G. W. accessed and verified the data. M. B., J. W., and L. J. B. were responsible for the decision to submit the manuscript.

***PROMISE investigators.*** Eline Harding, Marie-Noëlle Billard, Joanne Wildenbeest, and Louis Bont (University Medical Center Utrecht); Andrew Pollard (University of Oxford); Ana Dacosta-Urbieta and Federico Martinón-Torres (Servicio Galego de Saúde); Terho Heikkinen (University of Turku and Turku University Hospital); Steve Cunningham, Kate Templeton, and Harish Nair (University of Edinburgh); Peter Openshaw (Imperial College); Philippe Beutels (Antwerp University); Hannah Nohynek (Finnish Institute for Health and Welfare); Anne Teirlinck (National Institute for Public Health and the Environment Netherlands); John Paget (Netherland Institute for Health Services Research); Leyla Kragten (the ReSViNET Foundation); Carlo Giaquinto (Fondazione Penta ONLUS); Javier Diez-Domingo (Fundacio per al Foment de la Investigacio Sanitaria i Biomedica); Rafael Mikolajczyk (Martin Luther University Halle-Wittenberg); Gael Dos Santos (GlaxoSmithKline); Tin Tin Htar (Pfizer); Jeroen Aerssens (Janssen); Charlotte Vernhes and Rolf Kramer (Sanofi Pasteur); Veena Kumar (Novavax); Bahar Ahani (AstraZeneca); Eva Molero (Team-It Research).

***Disclaimer.*** This manuscript reflects only the views of the authors. The European Union and the Innovative Medicines Initiative are not responsible for any use that might be made of the information it contains.

***Data availability.*** Data are available upon request to the PROMISE (Preparing for RSV Immunization and Surveillance in Europe) Consortium (https://imi-promise.eu/).

***Financial support*.** This project received funding from the Innovative Medicines Initiative 2 Joint Undertaking (grant agreement number 101034339), with support from the European Union's Horizon 2020 research and innovation program and the European Federation of Pharmaceutical Industries and Associations.
