# Supplementary material for: Inconsistent Increase in Age at Respiratory Syncytial Virus Hospitalization of Children Aged <2 Years During the Severe Acute Respiratory Syndrome Coronavirus 2 Pandemic: A Retrospective Multicenter Study in 4 European Countries
Source: J Infect Dis. 2024 Jun 24;230(5):e985–95. doi: 10.1093/infdis/jiae292 (PMC11566234; doi:10.1093/infdis/jiae292)
Supplement: jiae292_Supplementary_Data [file jiae292_supplementary_data.docx]

**Supplementary material**

**Supplementary table 1. RSV testing practices for hospitalised children <2 years per site.**

|  | **Season** | **2017-2018** | **2018-2019** | **2019-2020** | **2020-2021** | **Summer 2021** | **2021-2022** | **Summer 2022** | **2022-2023** |
| --- | --- | --- | --- | --- | --- | --- | --- | --- | --- |
| **The Netherlands** | **RSV testing policy** | All admissions <2 years with respiratory symptoms | All admissions <2 years with respiratory symptoms | All admissions <2 years with respiratory symptoms | All admissions <2 years with respiratory symptoms | All admissions <2 years with respiratory symptoms | All admissions <2 years with respiratory symptoms | All admissions <2 years with respiratory symptoms | All admissions <2 years with respiratory symptoms |
|  | **RSV tests** | PCR or molecular POC test | PCR or molecular POC test | PCR or molecular POC test | PCR | PCR | PCR | PCR | PCR |
| **Spain** | **RSV testing policy** | All admissions <2 years with respiratory symptoms | All admissions <2 years with respiratory symptoms | All admissions <2 years with respiratory symptoms | All admissions <2 years with respiratory symptoms | All admissions <2 years with respiratory symptoms | All admissions <2 years with respiratory symptoms | All admissions <2 years with respiratory symptoms | All admissions <2 years with respiratory symptoms |
|  | **RSV tests** | PCR (unless Ag test positive in the ED) | PCR (unless Ag test positive in the ED) | PCR (unless Ag test positive in the ED) | PCR | PCR. | PCR. | PCR. | PCR. |
| **Scotland** | **RSV testing policy** | All admissions <2 years with respiratory symptoms | All admissions <2 years with respiratory symptoms | All admissions <2 years with respiratory symptoms | All admissions <2 years with respiratory symptoms | All admissions <2 years with respiratory symptoms | All admissions <2 years with respiratory symptoms | All admissions <2 years with respiratory symptoms | All admissions <2 years with respiratory symptoms |
|  | **RSV tests** | PCR or molecular POC test | PCR or molecular POC test | PCR or molecular POC test | PCR or molecular POC test | PCR or molecular POC test | PCR or molecular POC test | PCR or molecular POC test | PCR or molecular POC test |
| **Finland** | **RSV testing policy** | All admissions <2 years with respiratory symptoms | All admissions <2 years with respiratory symptoms | All admissions <2 years with respiratory symptoms | All admissions <2 years with respiratory symptoms | Unknown | All admissions <2 years with respiratory symptoms | Unknown | All admissions <2 years with respiratory symptoms |
|  | **RSV tests** | PCR (unless Ag test positive in the ED) | PCR (unless Ag test positive in the ED) | PCR (unless Ag test positive in the ED) | PCR test | Unknown | PCR test | Unknown | PCR test |

Table 2. Testing practices per site per season. 2017-2018, 2018-2019, 2019-2020, 2020-2021, 2021-2022 and 2022-2023 were defined from October to April. The summer of 2021 and 2022 were defined from May to September. Abbreviations: PCR = polymerase chain reaction; POC = point of care test; Ag-test = Antigen test; ED = Emergency Department.

**Supplementary table 2. List of included ICD-10 codes for the case definition of ARI and RSV hospitalisations.**

| **ARI ICD-10 codes** | J00 J02.0 J02.8 J02.9 J03.0 J03.8 J03.9 J04.0 J04.1 J04.2 J05.0 J05.1 J06.0 J06.8 J06.9 J09 J10.0 J10.1 J10.8 J11.0 J11.1 J11.8 J12.0 **J12.1** J12.2 J12.3 J12.8 J12.9 J13 J14 J15.0 J15.1 J15.2 J15.3 J15.4 J15.5 J15.6 J15.7 J15.8 J15.9 J16.0 J16.8 J17.0 J17.1 J17.2 J17.3 J17.8 J18.0 J18.1 J18.2 J18.8 J18.9 J20.0 J20.1 J20.2 J20.3 J20.4 **J20.5** J20.6 J20.7 J20.8 J20.9 **J21.0** J21.1 J21.8 J21.9 J40 J22 |
| --- | --- |
| **RSV ICD-10 codes** | **J12.1 J20.5 J21.0 B974 (in the presence of a J code )** |
